# The Promise of Disease Detection Dogs in Pandemic Response: Lessons Learned From COVID-19

**DOI:** 10.1017/dmp.2021.183

**Published:** 2021-06-08

**Authors:** Cynthia M. Otto, Tara Kirk Sell, Tener Goodwin Veenema, Divya Hosangadi, Rachel A. Vahey, Nancy D. Connell, Lois Privor-Dumm

**Affiliations:** 1 University of Pennsylvania School of Veterinary Medicine, Philadelphia, Pennsylvania, USA; 2 Johns Hopkins Center for Health Security, Baltimore, Maryland, USA; 3 Department of International Health, Johns Hopkins Bloomberg School of Public Health, Baltimore, Maryland, USA

**Keywords:** scent detection dogs, COVID-19, pandemic, public health, policy

## Abstract

One of the lessons learned from the coronavirus disease 2019 (COVID-19) pandemic is the utility of an early, flexible, and rapidly deployable disease screening and detection response. The largely uncontrolled spread of the pandemic in the United States exposed a range of planning and implementation shortcomings, which, if they had been in place before the pandemic emerged, may have changed the trajectory. Disease screening by detection dogs show great promise as a noninvasive, efficient, and cost-effective screening method for COVID-19 infection. We explore evidence of their use in infectious and chronic diseases; the training, oversight, and resources required for implementation; and potential uses in various settings. Disease detection dogs may contribute to the current and future public health pandemics; however, further research is needed to extend our knowledge and measurement of their effectiveness and feasibility as a public health intervention tool, and efforts are needed to ensure public and political support.

The coronavirus disease 2019 (COVID-19) pandemic has highlighted a critical need for improved preparedness for future pandemic threats. Importantly, the largely uncontrolled spread of the pandemic within the United States has exposed a range of planning and implementation shortcomings which, if they had been remedied before the pandemic emerged, could have significantly changed the trajectory of the outbreak in this country. One critical gap that has been highlighted is the need for a flexible and rapidly deployable disease screening and testing response.

In the weeks and months following the emergence of COVID-19, countries have struggled to establish rapid and widescale disease screening and testing. Early in the pandemic, the only people who could receive COVID-19 testing were those who had recently traveled, preventing the identification of domestic transmission and delaying critical public health responses. Further complicating the response was the realization that asymptomatic transmission was quite high, now shown to be found in more than half of COVID-19 cases.^1^ Had rapid screening been deployed at potential hotspots early in the outbreak, the public health response could have been initiated sooner, possibly limiting the need for more extreme disease containment measures later on. Rapid screening using antigen testing^2^ is one such method that is being implemented almost a year into the spread of the infection. We explore the use of disease detection dogs as one component of a flexible, rapidly deployable screening and testing system. Use of these dogs during the COVID-19 pandemic has already demonstrated potential in detecting disease, but their capabilities must be further investigated, validated, and expanded to be of use in this and future pandemics.

Although disease detection dogs are not a replacement for traditional screening and diagnostic testing and there are limitations to their use, they have advantages as a potential noninvasive screening approach that is immediate, efficient, reagent-free, and may be more cost-effective than other forms of diagnostic testing.

## Existing Evidence for Disease Detection Dogs

The use of dogs in detection of volatile organic compounds (VOCs), which are emitted through skin, breath, and bodily fluids, is not new.^3–5^ However, the approach has not yet been used on a widespread basis for detection or mass screening in the setting of a global pandemic. Dogs have been trained and used for detection of drugs and explosives on either articles or people in airports or other locations and are used by government airport security, law enforcement agencies, and private companies throughout the world. Dogs have also been used in search and rescue operations and have been proven to be an invaluable tool to detect the scent of live or deceased humans. For medical detection, they have been used in multiple capacities, including assistance dogs (e.g., alerting for drops in blood sugar [hypoglycemia] in diabetics or onset of seizures).

Several diseases, such as Parkinson disease^6^; bacterial^3,7^ and viral, including influenza, infections^8,9^; and several types of cancer^10–14^ have been documented to have unique VOC profiles by either chemical sensors (gas chromatography, mass spectrometry)^4^ or biological sensors (dogs).^15^ Dogs can detect VOC signatures through their sense of smell of different biological fluids, such as urine,^16^ saliva,^17^ sweat,^18^ and breath samples.^5^ One study in type 1 diabetes showed that the dogs, when trained on limited samples, did not respond to hypoglycemia in new samples on which they were not trained.^19^ Although the sensory capability of dogs is well known, the training of the dogs to identify an odor that is specific to the disease of interest, but still generalize the odor across a wide variety of individuals is a challenge.^20^ Due to requirements for sufficient numbers of both positive and appropriate control samples, the necessary diversity of samples to represent the population to be screened, the appropriate handling and storage of samples to preserve the odor, prevent disease transmission and avoid sample contamination, there exists a need to standardize research protocols and conduct regular double blind testing to determine each dog’s sensitivity and specificity for the disease of interest.^15,20,21^

## Evidence for SARS-COV-2 Detection Dogs During the COVID-19 Pandemic

VOCS emitted by people infected with severe acute respiratory syndrome coronavirus 2 (SARS-CoV-2), the causative agent of COVID-19, have characteristics that are distinguishable from those released by uninfected individual.^22,23^ Specially trained dogs have been able to detect SARS-CoV-2–specific VOCs and distinguish them from those released by other diseases.^24^ Proof of concept studies have been conducted showing high degrees of “success.”^17,18^ This ability to sniff the VOCs produced by COVID-19–positive individuals provides a promising approach to detection of COVID-19 cases or other diseases in certain settings. Additionally, specially trained COVID-19 detection dogs have demonstrated an ability to detect presymptomatic and asymptomatic patients who are less likely to be screened, given that many do not suspect that they are infected.^18,24^

Although reverse transcriptase polymerase chain reaction (RT-PCR) testing is considered the gold standard in diagnostic testing for COVID-19, studies have found varying sensitivity and specificity depending on testing protocols; at least 1 study found a high rate of false negatives in presymptomatic people.^25^ While several considerations must still be understood and addressed, the use of specially trained COVID-19 detection dogs offers the potential for a rapid and noninvasive detection of disease. The use of dogs may potentially provide a greater degree of public acceptance due to its noninvasive and convenient approach, removing the need for appointments and the need to remove a mask for RT-PCR testing requiring samples taken by means of nasal swab at specially designated locations, hospitals, or commercial laboratories.

There have been several proof-of-concept studies published describing the use of scent dogs in detection of SARS-CoV-2. Evidence from 2 trials demonstrated that dogs could detect the virus from saliva and tracheobronchial^17^ and sweat^18^ samples, respectively. In the first double-blind, randomized controlled trial at University of Veterinary Medicine Hannover, in Germany, 8 dogs were trained to accurately detect SARS-CoV-2 in 1012 samples of 7 positive and 7 negative hospitalized patients. The dogs had an overall detection rate of 94% with 83% (95% confidence interval [CI]: 82-84%) sensitivity and 96% (95% CI: 96.3-96.4%) specificity^17^; however, the statistical power of this study was low with limited sample numbers and a failure to confirm whether the dogs could generalize the odor and detect samples from novel patients.

Another study conducted in 2 locations (Alfort School of Veterinary Medicine in Paris, France, and French Lebanese University St. Joseph in Beirut, Lebanon) trained 14 dogs, including explosive, search and rescue, and colon cancer detection dogs on sweat samples from the underarms of COVID-19–positive and -negative hospitalized patients.^18^ Safety of the dogs and humans was considered in the study and investigators found no evidence of transmission in either direction or any symptoms in the dogs.^17^ Six dogs completed both training and detection trials and were able to detect SARS-CoV-2 accurately in samples on which they had not previously trained.^17^ The success rate per dog (ie, the number of correct indications divided by the number of trials) ranged from 76% to 100%. Two samples initially deemed negative by the hospital were identified as positive by the dogs were later confirmed to be positive, demonstrating the potential for dogs to detect disease earlier than standard PCR tests.^17^ In a report published by Nature, dogs screened sweat samples of 1680 arriving passengers in the airport in Lebanon and found 158 COVID-19 cases that were confirmed by PCR tests. The animals correctly identified negative results with 100% accuracy, and correctly detected 92% of positive cases.^22^ While suggesting that there is variability in performance by the dogs, the study indicated that a well-trained dog could detect SARS-CoV-2.

Additional studies conducted in other locations including United Arab Emirates^23^ reported canine sensitivity of detection ranging from 92% to 98% in sweat samples; similar to results in the French study, dogs detected presymptomatic cases not initially picked up by PCR.^24^ Additional studies are ongoing in several other locations including at University of Pennsylvania Working Dog Center,^26^ the United Kingdom,^27^ Belgium, Australia, Argentina, Brazil, and Chile.^28^ The Helsinki airport used 2 specially trained dogs to detect VOCs for COVID-19 in airport passengers, reporting nearly 100% accuracy for detection of COVID-19 in samples collected.^29^ While further studies are needed to standardize the approach and validate results, including sensitivity and specificity, as discussed below, these studies are encouraging.

## Research Agenda

Although disease detection dogs are currently in use in a small number of settings, more data are needed to provide valid and reliable evidence to support the widespread use of specially trained dogs in surveillance and detection of pathogens. There are several gaps in our knowledge related to the training and deployment of medical detection dogs.^20,24^ The need for a large number of samples for training and testing is often a limiting factor. Research is needed to determine the optimal number and diversity of samples for training and testing. Until further research is conducted, current recommendations on the best practices in conducting canine medical detection research should be followed.^20^

### Specific Training Needs

- Sample source: the samples must be obtained from a diversity of people with varying ethnicity, age, gender, concurrent illness that represent the population to be screened.
- Positive and negative samples for training require confirmation of the disease state; this may be confounded by false negative or false positive tests used as the gold standard.
- Negative samples should be from individuals with symptomology similar to the disease in question (e.g., if training for SARS-CoV-2, samples from individuals with influenza and common cold are needed).
- Positive samples should be from those with symptoms consistent with the population to be screened (e.g., for SARS-CoV-2, include asymptomatic or mildly affected individuals).
- The number of samples for training should be in excess of 100 positives for initial training and collection of new samples are required for testing and maintenance training.
- The samples should be stored and handled in a way that preserves the odor and prevents contamination.
- The number of negative samples should reflect the prevalence in the population. If 10% of people are affected, there should be 9 negatives for each positive.
- Dogs should be trained in a way that they do not expect to always find a positive sample.
- Training should be conducted in a way that reflects the operational usage: initial training may require scent boxes/scent wheels, but if dogs are to be screening people, training should be done with people.
- The alert to odor should be consistent, reproducible, clearly defined, and consistent with the operational usage (eg, barking or scratching would not be acceptable when screening people).
- Dogs need to have objective and regular performance assessments that include double blind testing with novel samples in an operational setting.
- Training protocols should be standardized.

Proper statistical tools should be used, including adjusting for testing multiplicity, repeated measures designs, and the probability of type I errors.

## Accuracy of Disease Detection Dogs

Determining the accuracy of a diagnostic or screening test requires evaluation of the specificity and sensitivity of the test. In general, the higher the value of these two characteristics are, the more accurate the test. Sensitivity is the ability of a test to correctly identify positive samples.

For disease detection dogs, the animal must recognize an individual with infection and bypass uninfected people. Specificity is the ability of a test to correctly identify samples that come from individuals that do not have the infection. Applying these testing standards to scent dog performance is challenging, because the number of dogs involved in research studies is low. Subtle differences in the method of sample processing can lead to artificially high performance. Guest et al. examined the use of scent dogs to detect prostate cancer in urine samples from afflicted patients.^21^ After training the dogs with prepared samples, performance was excellent (mean sensitivity 93.5% and specificity 87.9%). But when performance was tested on hospital-based samples, specificity dropped precipitously 67.3% (43.2-83.3). The dogs were able to distinguish between the two different processes involved in the sample preparation. This careful work illustrates the care with which these studies must be designed to eliminate systematic differences, especially if the samples are as complex and variable as breath or clothing and the disease has a spectrum of symptoms.

Thus, determining the validity of a test requires consistent standards. For example, in the case of COVID-19 detection, it is crucial that we determine that the dogs are detecting the specific VOC(s) associated with SARS-CoV-2 infection and not something else. Efforts have been made to identify sensitivity and specificity, but it would still need to be ascertained by type of sample and in a variety of people from different demographics with different symptoms. Furthermore, canines exhibit variability in behavior and performance from day to day. It is possible that they may miss some positive people and for that reason it is important to build redundancy into the system.

## Operationalizing the Use of Disease Detection Dogs

In light of a growing evidence base supporting that disease detection dogs can achieve adequate forecasting accuracy for determinations that disease is present and for determinations that disease is not, there are several operational and logistical considerations that may impact feasibility (Table 1). First, the supply of properly trained healthy dogs must be sufficient. Assuming, for a particular public venue, that a minimum of two dogs would be needed to allow the dogs to work for 20-30 min and rest for 20-30 min, a pair of dogs could potentially screen up to 250 people per hour. According to the veterinary literature, certain general traits, such as high play motivation, cooperativeness with their handlers, obedience yet independence, and more have been identified as characteristics in dogs suitable for this type of work.^30^ Identifying a way to both select appropriate dogs and their human trainers will be important. Use of disease detection dogs that have already been trained may speed up training^31^; however, the use of dogs for example, previously trained on explosives may result in a risk that the dog’s alert could reflect an explosive threat rather than the current disease odor.

**Table 1.** Implementation needs

Early implementation (proof of concept)	Widespread use
• Supply of dogs (ideally professionally or privately owned dogs trained on odor) • Veterinarian support for care of dogs • Supply of trainers, equipment • Standards of measurement and safety • Access to diverse samples for research • Funding for research, training, acquiring dogs	• Technical consensus for use case (see below) • Investment case to determine resources required and impact • Supply of dogs and trainer determined to have high likelihood of success for use case – (see below) • Political will (eg, support from government to implement dog detection programs) • Monitoring and evaluation • Funding for all of the above • Veterinary oversight to protect health and well-being of the dogs • Community engagement, education

Currently, there are a limited number of centers globally that train working dogs for medical detection. Although there are voluntary programs that provide certification for service dogs (e.g., Assistance Dogs International) and police dogs (e.g., United States Police Canine Association, North American Police Work Dog Association), there are no organized accrediting associations for medical detection dogs and their trainers. There are also no universal or mandated performance standards for detection dogs; however, the National Institute of Standards and Technology (NIST Dogs & Sensors Subcommittee)^32^ is in the process of publishing standards. An accrediting organization could support an industry that could create jobs and add further validity to a cadre of trainers and handlers throughout the world. Furthermore, the greater the demand for trainers and specially trained detection dogs, the more streamlined and cost-effective this effort could become. The more prominent the effort and its affiliation with research institutions, the stronger the science, the better the training, and the easier it will become to obtain samples for research.

## Economics of Disease Detection Dogs

It will be important to develop an investment case to outline how a disease detection dog program would be cost-effective and feasible as a public health intervention. One potential option for planning is to incorporate use of disease detection dogs as part of nonpandemic medical screening and rapidly redeploy dogs during an outbreak. The cost of disease detection dogs can vary substantially. The Transportation Security Administration (TSA) reports start-up costs of training bomb-sniffing dogs to be over $200,000 and then more than $150,000 thereafter including the cost of the handler, veterinary care, food, and certification.^33^ Another TSA estimate for training a passenger screening dog and handler was $42,000.^34^ Other lower estimates take into account the cost to acquire a dog ($10,000) and $16,000 for scent detection school. Costs for personnel and care would be figured separately.^35,36^ Estimating that a dog could sniff 250 people per hour, working a 6-hour day for 30 min on, 30 min off, 5 days/week, a single dog could screen more than 189,000 people in a year. The cost per person for screening could range from 13.7 cents (for training and dog acquisition) to $1.05 (if fully costed start-up). Although this is not low cost, approximately $200M when considering the full start-up costs based on a 1000 dog/handler teams, similar to what is already used by TSA for airports, mass transit, and maritime systems,^34^ expenditures would likely decline as there become economies of scale. Once a dog is trained to detect one scent, it could potentially be trained to detect others, increasing utility and cost-effectiveness.

## Appropriate Settings

Detection dogs will have value to public health across settings with high risk of serious outcomes from the disease, such as rapid screening of residents and staff in long-term care facilities in the case of SARS-CoV-2. They also include settings where the risk of disease transmission is high, such as in prisons, food processing, or manufacturing, where there is limited ability to reduce population density. Other key settings include supporting operation of essential services, such as public transport, police, fire, health, and education.

Disease detection dogs are most likely to be an advantage in large congregate settings where many people must be screened in a short amount of time and in a physical environment where people routinely move through central checkpoints, such as in airports or K-12 schools where large numbers of people are moving or can be directed to move through a location in an organized manner. The identification of a potentially positive case must be accurate and actionable. Authorities must be able to document those noted in the screening process and direct them to definitive testing services. It is unlikely that disease detection dogs will provide the sole information required for final decisions. Rather, they will indicate where follow-up testing is needed.

There are multiple examples where disease detection dogs could be used, to make an impact in both the public and private sector. The criteria for use of dogs could be defined based on the considerations shown in Table 2.

**Table 2.** Use of disease detection dogs in outbreak response; considerations for allocation of scarce resources

	Number of facilities	High risk of serious outcome	High transmission risk not easily mitigated	Physical checkpoints feasible	Actionable (direct toward testing)	Throughput potential
Vulnerable populations^a^	15,600	x		x	x	+
Schools or universities^b^	209,298		x	?	x	++
Public transit	6800 organizations (APTA)		x	x	?	+++
Grocery stores	40,000	?	?	x	?	++
Manufacturing			x	?	?	++
Airports	5080 (public)	?	x	x	x	+++
Migrant crossings	330 (US-Mexico border)	?	x	x	x	++

Abbreviation: APTA, American Public Transportation Association.

a In the case of COVID-19, congregate settings such as long-term care facilities.

b School breakdown: K-12 schools (130,000-NCES 2017-18 data), public high schools (24,000), private secondary schools (16,000), combined schools (35,000), universities (4298) (2017 data from https://nces.ed.gov).

Decisions about where to use disease detection dogs should also be informed by community leaders who can provide input on practical considerations regarding acceptance of the dogs by the community. Acceptance of working dogs, which may already be in use in certain situations or be deemed too scary for other populations, is important. The presence of a dog “industry”—breeders, trainers, or presence of schools of veterinary medicine—may also play a role in feasibility. Dogs will need to be specially selected, so a supply of dogs with particular characteristics that make them good candidates for training can be derived from breeders or from shelter dogs,^37^ but if the latter, a good method of characteristic screening is essential because evaluating shelter dogs for working detection careers can be challenging.^38^

## Future Research

Although the use of disease detection dogs has clearly shown promise in certain settings, additional research is needed to ensure that the dogs are able to detect disease across large numbers of people. Dogs have been trained on samples from hospitalized patients; but in real-world settings, to harness the true power of the nose, they will need to detect scents in a public setting and ideally at a distance of 6 feet from the dog. It will be important to prove that the dogs can differentiate affected from unaffected with a measured level of sensitivity and specificity for multiple diseases. To ensure the dogs can accomplish these tasks, it will need to be known when the VOC(s) is produced during the course of infection and how long the scent lingers, whether there are any racial or ethnic factors that influence the odor and whether that odor can spread to nearby individuals. Finally, the impact of the operational environment, noise, air flow, temperature, humidity, ambient odors, and disease prevalence must all be considered.

Despite the potential of the SARS-CoV-2 detection dog program, there is little consistency in technological methodologies used for training, accuracy determination, and sample and control preparation; standards for these methods as well as monitoring and oversight of implementation will be required. The safety of the dogs and the humans involved in the screening will need to be ensured. It has been shown that dogs are more resistant to infection with SARS-CoV-2 than other species,^39^ but the virus has been recovered from a limited number of dogs, and dogs have developed antibodies to the virus.^40^ Safety precautions are necessary when using biological samples, virus inactivation or selection of samples that do not support active virus are critical for training and testing. If live humans are being screened, proper protective equipment for the handlers and the persons being screened is essential. The use of SARS-CoV-2 detection dogs would require technical consensus on the science, safety, and feasibility of the dogs, and agreement on regulatory pathways and oversight. Currently, there are no agencies overseeing standards for scent detection work, and research will be required to identify best practices and determine who would oversee such efforts. Finally, the dogs and people should be monitored for exposure to the virus. The susceptibility of dogs to future pandemic agents will also impact the role of dogs in detection of those agents. A quality control plan and standards for trainers, dogs, and handlers with a single organization or agency identified to ensure quality assurance is needed.

Screening for disease will likely be useful in very early stages of a pandemic, as retraining of experienced dogs can be quite rapid. Importantly, the approach can be focused during “peacetime” on targeted outbreaks. This public health intervention is unique in that it will require engagement and collaboration of those involved in both human and veterinary health. The SARS-CoV-2 detection dog program is consistent with the One Health initiative sponsored by the World Health Organization and the Centers for Disease Control and Prevention.^41^ One Health is a collaborative, multisectoral, and transdisciplinary approach—working at the local, regional, national, and global levels—with the goal of achieving optimal health outcomes by recognizing the interconnection between people, animals, plants, and their shared environment.

Moving forward with disease detection dogs will require the following action steps:

1. *Support for research to understand the potential and limitations of disease detection dogs during pandemic response.* Technical consensus and a more complete evidence base for use of disease detection dogs in during pandemics is needed.
2. *Establishment of standards for training and the establishment of sentinel training sites.* Standards for training and evaluation, as well as guidelines for use and oversight will be important to ensure that dogs are detecting disease with a high degree of reliability.
3. *Identification of optimal operational sites for the use of disease detection dogs.* The identification of appropriate settings will optimize the impact the use of disease detection dogs will have in pandemic responses.
4. *Show return on investment in other settings.* Use of detection dogs for diseases such as *Clostridium difficile, Candida auris*, influenza, and cancer could help establish use case and protocols as well as establish a readily deployable cadre of dogs for use in a future pandemic.

Disease detection dogs are a promising strategy for pandemic preparedness in addition to addressing screening gaps in the current pandemic. To incorporate this potential resource in combating COVID-19 and future pandemics, more research is needed as well as government and community investment into the infrastructure to implement and monitor the effectiveness of this approach.
